# Flucofuron as a Promising Therapeutic Agent against Brain-Eating Amoeba

**DOI:** 10.1021/acsinfecdis.4c00062

**Published:** 2024-05-17

**Authors:** Javier Chao-Pellicer, Iñigo Arberas-Jiménez, Ines Sifaoui, José E. Piñero, Jacob Lorenzo-Morales

**Affiliations:** † Instituto Universitario de Enfermedades Tropicales y Salud Pública de Canarias, Universidad de La Laguna, Avda. Astrofísico Fco. Sánchez, S/N, 38203 San Cristóbal de La Laguna, Spain; ‡ Departamento de Obstetricia y Ginecología, Pediatría, Medicina Preventiva y Salud Pública, Toxicología, Medicina Legal y Forense y Parasitología, Universidad de La Laguna, 38203 San Cristóbal de La Laguna, Spain; § Centro de Investigación Biomédica en Red de Enfermedades Infecciosas (CIBERINFEC), Instituto de Salud Carlos III, 28220 Madrid, Spain

**Keywords:** *Naegleria fowleri*, chemotherapy, Global Health Priority Box, flucofuron, programmed cell death

## Abstract

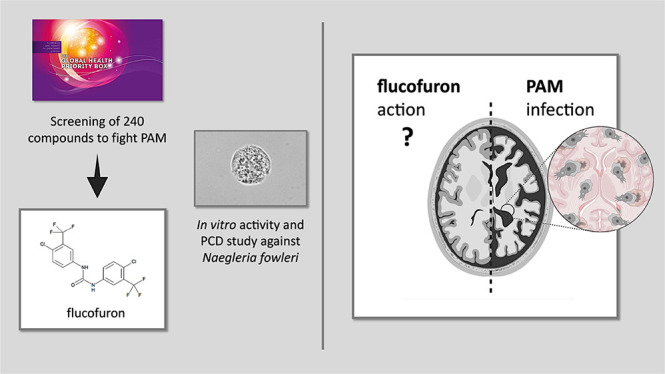

Primary amoebic meningoencephalitis (PAM) is a rare and fulminant neurodegenerative disease caused by the free-living amoeba *Naegleria fowleri*. Currently, there is a lack of standardized protocols for therapeutic action. In response to the critical need for effective therapeutic agents, we explored the Global Health Priority Box, a collection of 240 compounds provided by the Medicines for Malaria Venture (MMV). From this pool, flucofuron emerged as a promising candidate, exhibiting high efficacy against trophozoites of both *N. fowleri* strains (ATCC 30808 IC_50_ : 2.58 ± 0.64 μM and ATCC 30215 IC_50_: 2.47 ± 0.38 μM), being even active against the resistant cyst stage (IC_50_: 0.88 ± 0.07 μM). Moreover, flucofuron induced diverse metabolic events that suggest the triggering of apoptotic cell death. This study highlights the potential of repurposing medications for treating challenging diseases, such as PAM.

A large number of pathogens, almost imperceptible to the human eye, have the potential to cause major diseases in humans, some of which are understudied but are attracting the interest of the scientific community as emerging threats. These include certain microorganisms known to cause infections of the central nervous system (CNS), which can be fatal if left untreated. Among this group of pathogens are the free-living amoebae, including the genus *Naegleria*, with *Naegleria fowleri* as the only known species that causes pathogenicity in humans.^1^*N. fowleri*, commonly referred to as the “brain-eating amoeba”, is an emerging pathogen causing a fulminant disease called primary amoebic meningoencephalitis (PAM).^2^*N. fowleri* is known as a thermotolerant organism (30–42 °C), commonly found in warm freshwater environments, such as lakes, rivers, and hot springs.^3,4^ Its presence in aquatic habitats poses a potential risk to individuals engaging in recreational water activities, being essential to understanding the pathogenicity of this microorganism and the development of effective strategies to combat its detrimental effects. In terms of its life cycle, this species could be found in three different phenotypes, including the trophozoite form used as the vegetative phase, the cyst phase for survival under adverse environmental conditions, and the flagellar phase for searching for new nutrients using flagella.^5^ As previously mentioned, it has the ability to go from living in the environment at high temperatures to infecting individuals under suitable conditions. The disease it causes, PAM, is a rare, rapidly progressive illness with a fatality rate of approximately 95% of the few over 400 reported cases.^6,7^ The infection occurs by the inhalation of trophozoites in contaminated freshwater or, less commonly, through the inhalation of cysts that, once reaching the nasal cavity, transform into the trophozoite stage.^8^ These trophozoites move toward the brain through a chemotactic gradient created by their attraction to certain neurotransmitters, such as acetylcholine or noradrenaline.^9^ Along their path to the brain, they cause lesions in various areas until they reach the brain, where they begin to degrade tissue, resulting in reversible damage.^10,11^

Exploring the mechanisms of invasion and proliferation by *N. fowleri* in the brain provides insights into potential points of intervention for therapeutic development and effective treatment management of this potentially lethal infection. Early symptoms of PAM could be like those of bacterial meningitis, including severe headache, fever, nausea, vomiting, and neck stiffness. As the infection progresses, individuals experience a rapid onset of neurological symptoms, such as confusion, seizures, hallucinations, and coma, leading to severe brain swelling and eventual death.^3,12^ Given the rapid progression and high fatality rate associated with *N. fowleri* infections, early detection and prompt treatment are critical to improving patient outcomes.

Therefore, fast and accurate methods are needed for the detection of *N. fowleri* in clinical samples. Although diagnostic tools may not always be fully effective, they rely on various methods from cerebrospinal fluid samples, including the detection of live or fixed trophozoites by Giemsa staining, the induction of trophozoites into different stages, such as cysts or flagellar forms, and the detection of parasite genetic material using polymerase chain reaction (PCR).^13−16^ In addition to these laboratory techniques, their combination with computed tomography provides a means of detecting lesions produced with the assistance of medical specialists.^17,18^

In recent years, some success has been shown with the administration of antifungal or antimicrobial agents, such as miltefosine, amphotericin B, or certain azoles, which demonstrate synergistic activity.^19^ Despite not being entirely effective, they often present high toxicity in vital organs, leading to irreversible side effects in those administered with them. In combination with these drugs, neuroprotective therapy based on corticosteroids or the induction of hypothermia is often applied to slow the disease. Consequently, the search for alternative treatment strategies against PAM remains imperative to mitigate the high fatality rates caused by this infection.

In recent years, the Global Health Priority Box, a curated collection of compounds provided by Medicines for Malaria Venture, has gathered significant attention for its potential in combating a spectrum of parasitic diseases owing to its diverse chemical composition and broad-spectrum activity against various protozoans. Hence, this study aimed to evaluate the activity of Global Health Priority Box against *Naegleria fowleri*.

## 1 Results

### 1.1 Screening of the Global Health Priority Box Compounds

In the initial screening phase, 240 compounds were systematically evaluated against *N. fowleri* (ATCC 30808) trophozoites and murine macrophage cell line J774A.1 (ATCC TIB-67). Among the selected candidates, flucofuron revealed to be a promising candidate for further studies, having achieved the required criteria.

### 1.2 Inhibition and Cytotoxic Effect of Flucofuron

**Figure 1:**
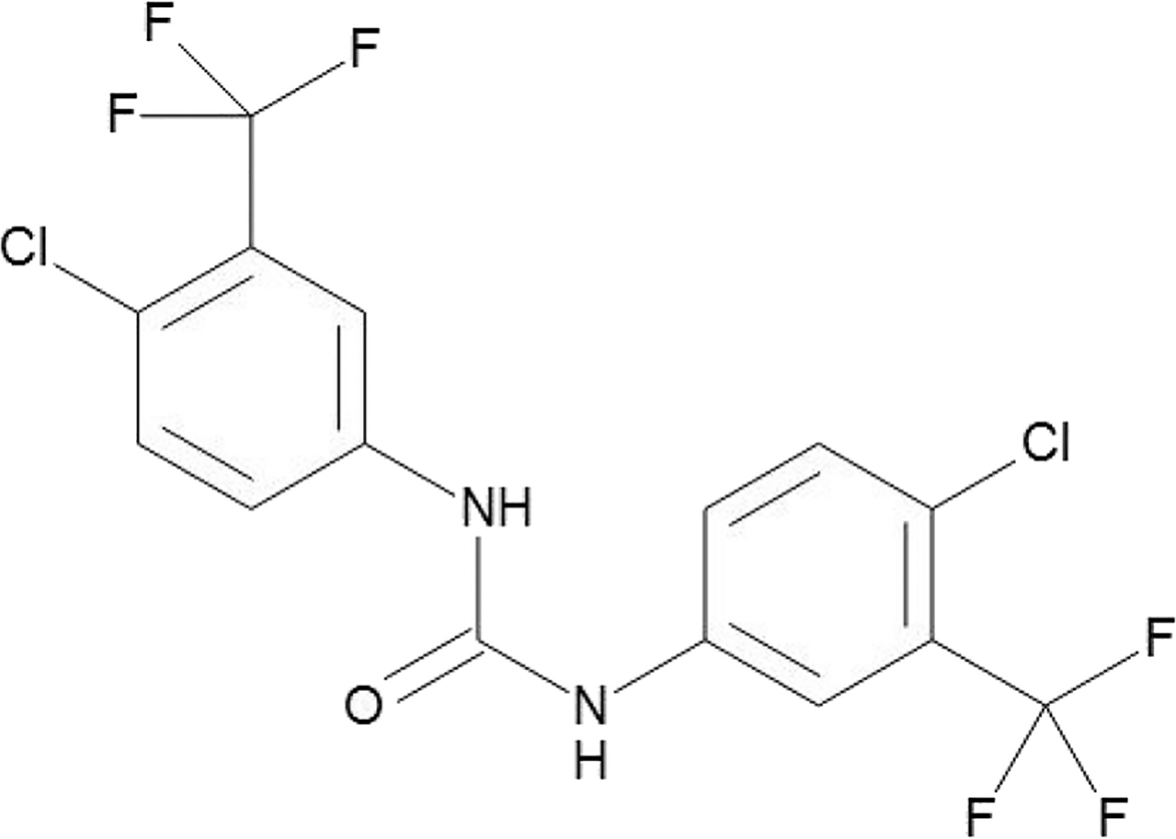
Flucofuron’s chemical structure.

The efficacy of flucofuron (Figure 1) against *N. fowleri* was determined using the colorimetric assay previously described. The compound exhibited inhibition values (IC_50_ ) against the ATCC 30808 and 30215 strains of 2.58 ± 0.64 and 2.47 ± 0.38 μM, respectively. Additionally, the cytotoxic concentration against murine macrophages, CC_50_, measured 83.86 ± 20.76 μM. The substantial activity relative to cytotoxicity emphasizes the high selectivity index values of 32.55 (ATCC 30808) and 33.96 (ATCC 30215) obtained. Moreover, compared to the trophozoite stage, flucofuron demonstrated an IC_50_ value of 0.88 ± 0.07 μM against the cysts, highlighting its lower effective concentration against the cyst stage.

### 1.3 Programmed Cell Death Induction

Diverse metabolic events were demonstrated in *N. fowleri* (ATCC 30808) trophozoites treated with flucofuron at 2.90 μM (IC_90_) for 24 h. Three experimental conditions were employed: treated amoebas with flucofuron and two control groups to evaluate the assay. Nontreated amoebae as the negative control and treated with IC_90_ amphotericin B as the positive control were used.

#### 1.3.1 Demonstration of Apoptotic Pathway Activation

##### 1.3.1.1 Chromatin Condensation Detection

**Figure 2:**
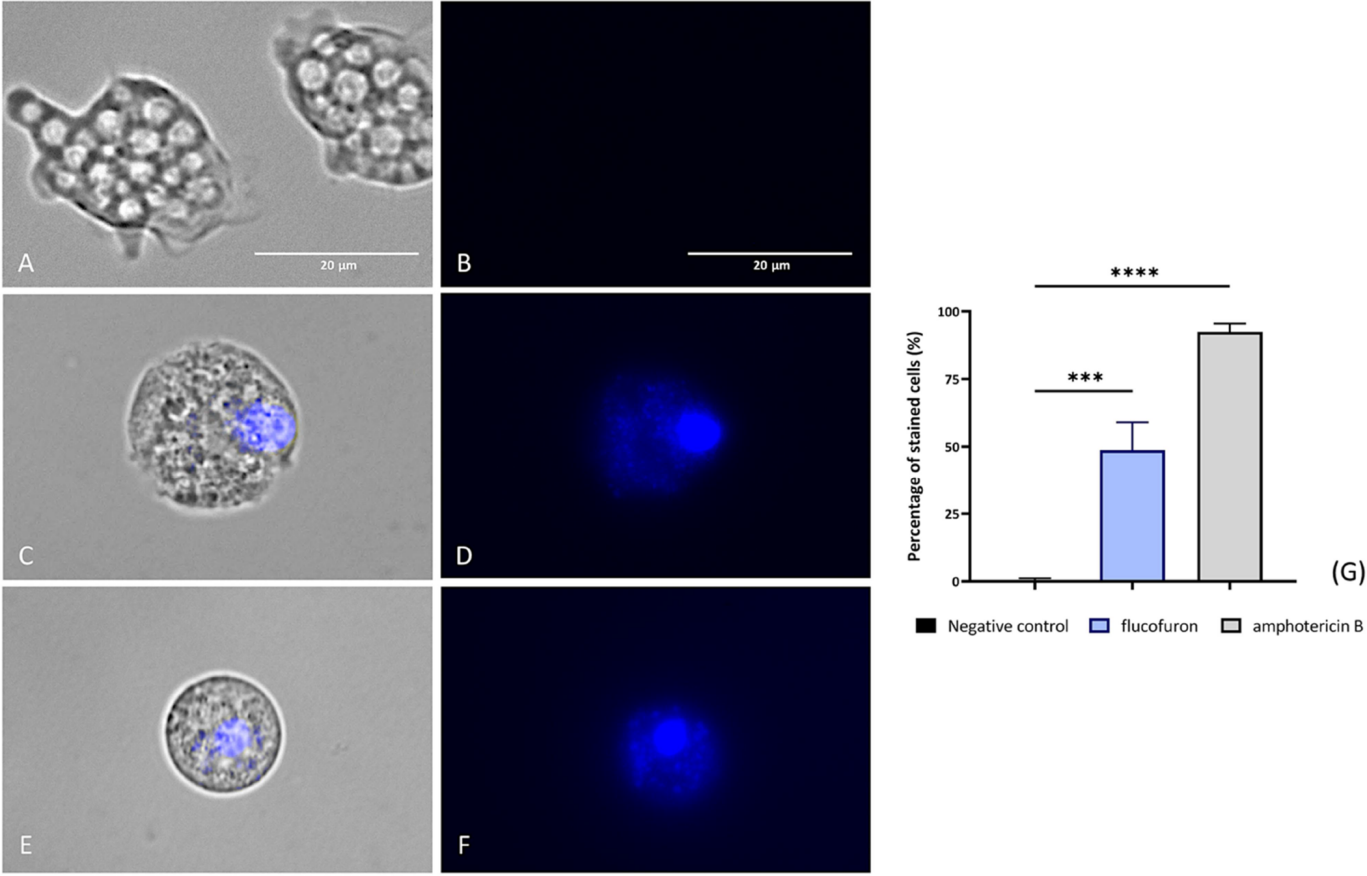
Accuracy of nuclear staining by Hoechst 33342 and PI staining. Nontreated amoebae were used as a negative control (A, B). Treated amoebae with flucofuron have been tested (C, D). Treated amoebae with amphotericin B served as a reference group (E, F). Treated amoebae with IC_90_ of chemicals (C–F) displayed blue fluorescence indicating the condensation of nucleus. This result was contrasted with an absence of fluorescence shown by negative control (A, B). Two different visible/fluorescent channels were simultaneously captured at 100× magnification using an EVOS M5000 Cell Imaging System, Life Technologies, Madrid, Spain. Scale bar: 20 μm. The bar graph (G) reports the percentage of stained cells emitting blue fluorescence. The data represent the average values of three separate assays, shown together with the standard deviation (SD). An ANOVA was also tested for statistical differences between treated cells and negative control, **** *p* < 0.0001; *** *p* < 0.001. Each count involved analysis of three different images of the cell population using EVOS M5000 software tools.

One characteristic early event in the apoptotic cell death pathway across organisms is chromatin compaction, a process detectable using Hoechst 33342 dye (Life Technologies, Madrid, Spain). This dye binds to chromatin condensation and emits blue fluorescence. The results obtained suggest chromatin condensation, as evidenced by the bright blue fluorescence emitted in amoebae treated with different drugs, amphotericin B (Figure 2D,E) and flucofuron (Figure 2G,H). These results were compared with the negative control group (Figure 2A–C), where fluorescence was absent, indicating a healthy state of these microorganisms. Differences between values were assessed using an ANOVA. The results displayed significant differences (**** *p* < 0.0001; *** *p* < 0.001) when comparing treated cells to the negative controls.

##### 1.3.1.2 Apoptotic and Necrotic Cell Differentiation

**Figure 3:**
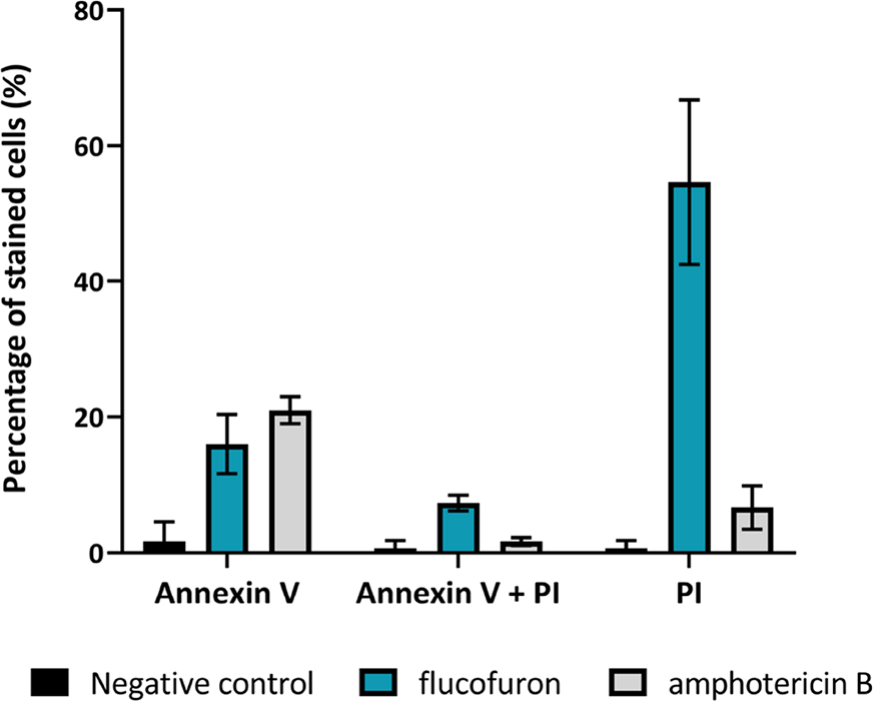
Histogram of stained cells with Annexin V Alexa Fluor 488 and PI. Detection of the apoptotic stage of amoebae under different experimental conditions. Nontreated, flucofuron-treated, and amphotericin B-treated trophozoites were the experimental groups, studying their viability at 24 h.

Cell viability of amoebae was determined using a double-stained apoptosis detection kit (Annexin V/PI) (Figure 3). The viability condition was differentiated into nontreated cells (negative control, black bars), amphotericin B-treated cells (positive control, gray bars), and flucofuron-treated cells (molecule tested, blue bars). At 24 h, 16 and 21% green fluorescence was emitted by Annexin v upon treatment with flucofuron and amphotericin B, respectively, indicating an early stage of apoptosis. In addition, under the same conditions, 7.3 and 1.67% were obtained in the presence of both fluorophores, indicating advanced processes of cell death. Finally, the 54.67% value of fluorescence in those treated with flucofuron compared to the 6.67% obtained with the positive control of red fluorescence emitted by PI, which would show values of dead cells after this time.

##### 1.3.1.3 Analysis of DNA Fragmentation

**Figure 4:**
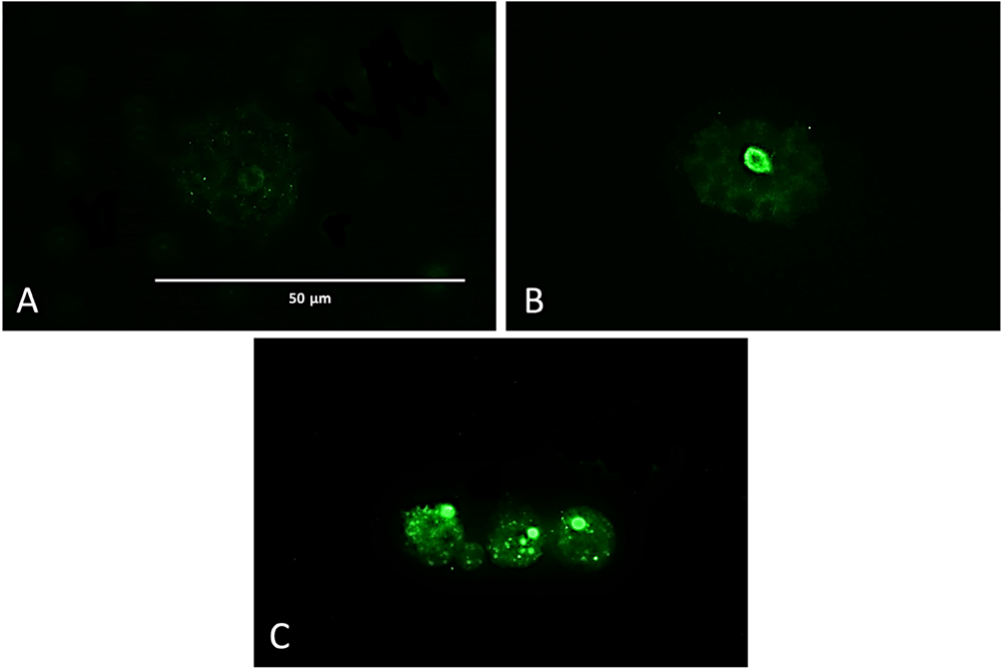
DNA fragmentation revealed by the TUNEL assay. Three different experimental conditions after 24 h. Negative control (A), using nontreated trophozoites. Positive control (B), trophozoites labeled with manufacturers’ DNase. Treated amoebae were treated with flucofuron IC_90_ (C). One fluorescent channel was captured at 63× magnification using an Echo Revolution hybrid automated microscope, Discover Echo, San Diego, CA.

The analysis of DNA fragmentation was performed by using the TUNEL assay. For this, trophozoites were subjected to different conditions. Two control groups, one without nontreated amoebae (Figure 4A) and another with DNase (Figure 4B) to demonstrate the validity of the study, and a group of trophozoites treated with the molecule under evaluation, flucofuron (Figure 4C). Treated cells (Figure 4B,C) displayed green fluorescence corresponding to DNA fragmentation. These results were compared with the ones obtained by the negative control group (Figure 4A), in the absence of fluorescence.

#### 1.3.2 Plasma Membrane Permeability

**Figure 5:**
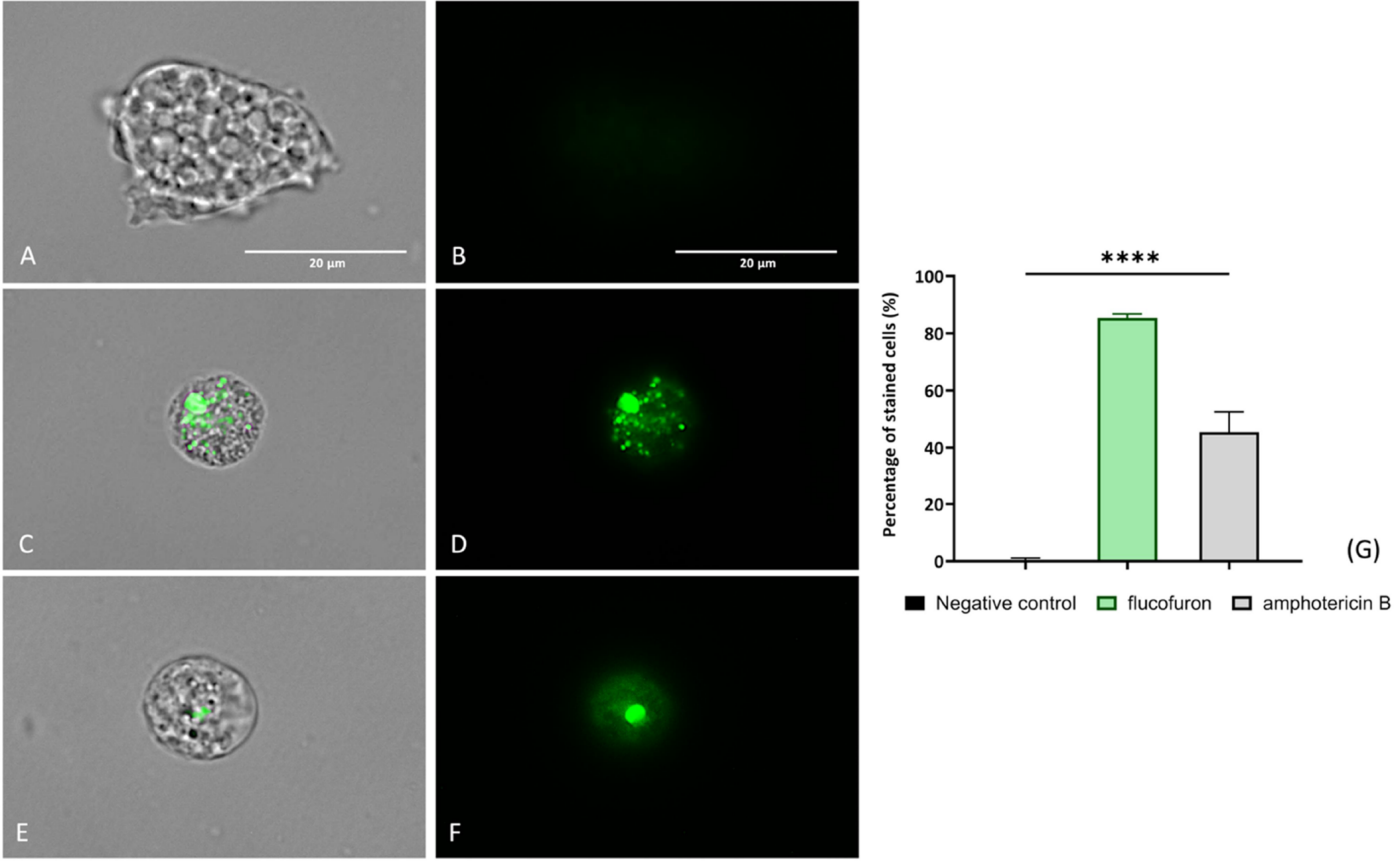
SYTOX Green reagent reveals plasma membrane alterations. Nontreated amoebae (A, B) were used as a negative control. Treated amoebae with flucofuron (C, D) have been tested. Treated amoebae with amphotericin B (E, F) served as a reference group. Treated amoebae with IC_90_ of chemicals (C–F) displayed green fluorescence, due to the permeabilization of the plasma membrane. This result was contrasted with an absence of fluorescence showed by negative control (A, B). Two different visible/fluorescent channels were captured simultaneously with a magnification of 100×. The EVOS M5000 Cell Imaging System, Life Technologies, Madrid, Spain, was employed to capture the images. Scale bar: 20 μm. The bar graph (G) reports the percentage of green-stained cells with the dye. The data represent the average values of three separate assays, shown together with the standard deviation (SD). An ANOVA was also tested for statistical differences between treated cells and negative control, **** *p* < 0.0001; ns, not significant. Each count involved analysis of three different images of the cell population using EVOS M5000 software tools.

To evaluate alterations in membrane permeability induced by the compound flucofuron, SYTOX Green dye (Life Technologies, Madrid, Spain) was employed. Trophozoites treated with flucofuron (Figure 5C,D) exhibited green fluorescence similar to our positive control (amphotericin B-treated cells; Figure 5E,F), suggesting plasma membrane damage and increased permeability. In contrast, the negative control (nontreated amoebae) (Figure 5A,B) remained impermeable to the reagent, resulting in no fluorescence emission. SYTOX Green targets the same cellular component as PI, binding to nucleic acids and causing fluorescence emission. However, SYTOX Green demonstrates greater sensitivity and efficacy in analyzing cell membrane permeability.^21^ This is evident in the green fluorescence observed in Figure 5, contrasting with the absence of red fluorescence in the previous assay using PI (Figure 2). An ANOVA was conducted to analyze disparities between mean values, revealing significant differences (****p < 0.0001) between the treated cells and the negative control.

#### 1.3.3 Mitochondrial Membrane Potential Depolarization

**Figure 6:**
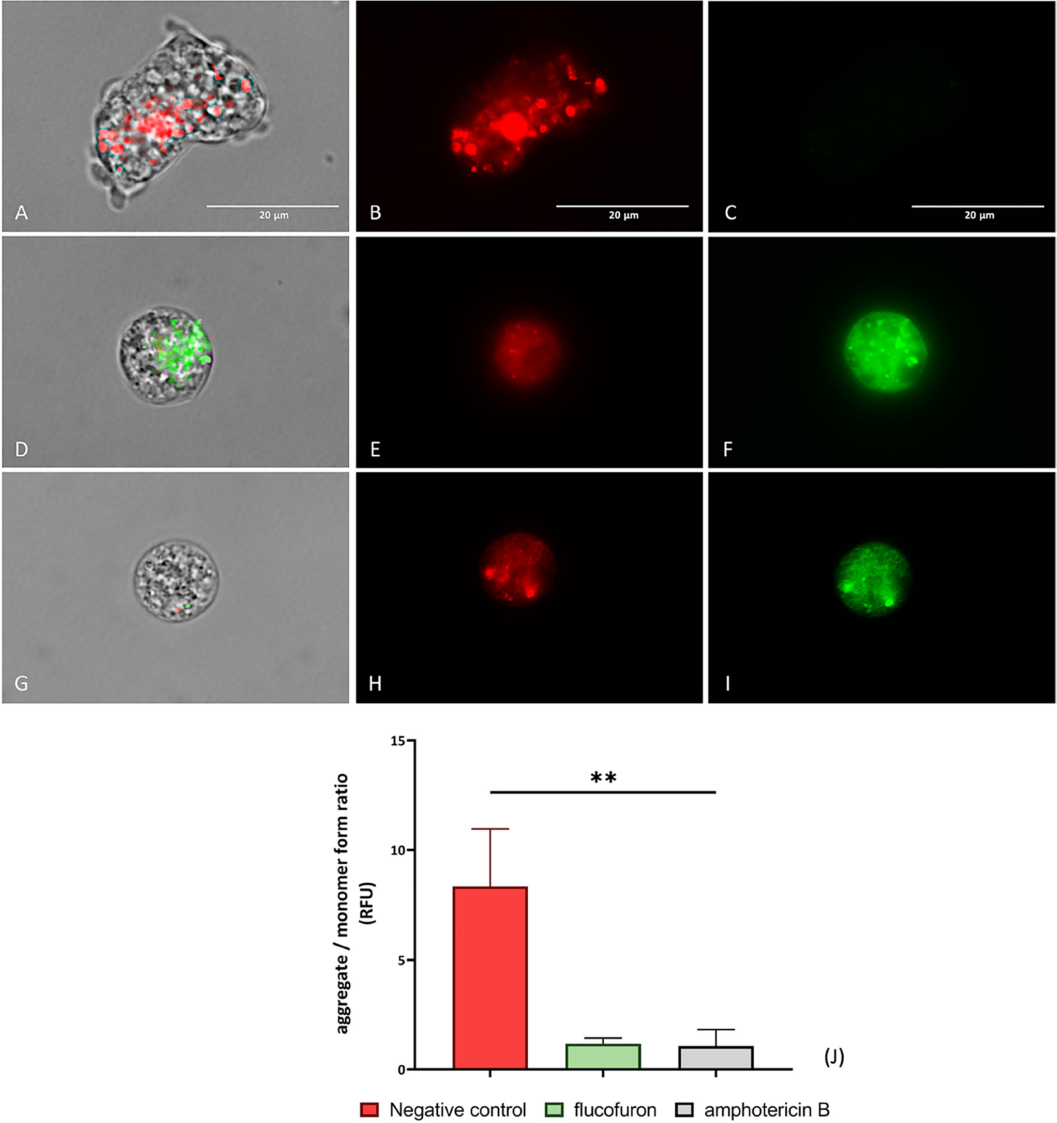
Mitochondrial membrane potential dipolarization was assessed by using JC-1. Nontreated amoebae (A–C) were used as a negative control. Treated amoebae with flucofuron (D–F) have been tested. Treated amoebae with amphotericin B (G–I) served as a reference group to validate the results. Treated amoebae (D–I) with IC_90_ of chemicals displayed green fluorescence due to the permeabilization of the plasma membrane. This result was contrasted with an absence of fluorescence showed by negative control (A, B). Three different visible/fluorescent channels were captured simultaneously with a magnification of 100×. The EVOS M5000 Cell Imaging System, Life Technologies, Madrid, Spain, was employed to capture the images. Scale bar: 20 μm. The bar graph (J) reports the ratio of aggregate (red)/monomer (green) form of JC-1. The data represent the average values of three separate assays, shown together with the standard deviation (SD). An ANOVA was also tested for statistical differences between treated cells and negative control, **** *p* < 0.0001; ns, not significant. Each count involved analysis of three different images of the cell population using EVOS M5000 software tools.

The analysis of the mitochondrial membrane potential dysfunction was assessed using a JC-1 Mitochondrial Membrane Potential Detection Kit (Cayman Chemicals Vitro SA, Madrid, Spain). In nontreated cells, observed in the negative control (Figure 5A–C), the reagent appears in the form of J-aggregates emitting red fluorescence, indicative of the positive charge in this organelle. In contrast, the exposure of amoebae to flucofuron (Figure 6D,F) leads to a significant decrease in this mitochondrial membrane potential, causing the reagent to disperse and adopt a monomeric form that emits green fluorescence. These results were consistent with those obtained from amoebae treated with flucofuron compared to the positive control, amphotericin B (Figure 6G–I), where green fluorescence emission was also emitted. ANOVA was performed to evaluate differences among the values, revealing significant distinctions (** *p* < 0.01) when comparing treated and nontreated cells.

#### 1.3.4 ATP Level Determination

**Figure 7:**
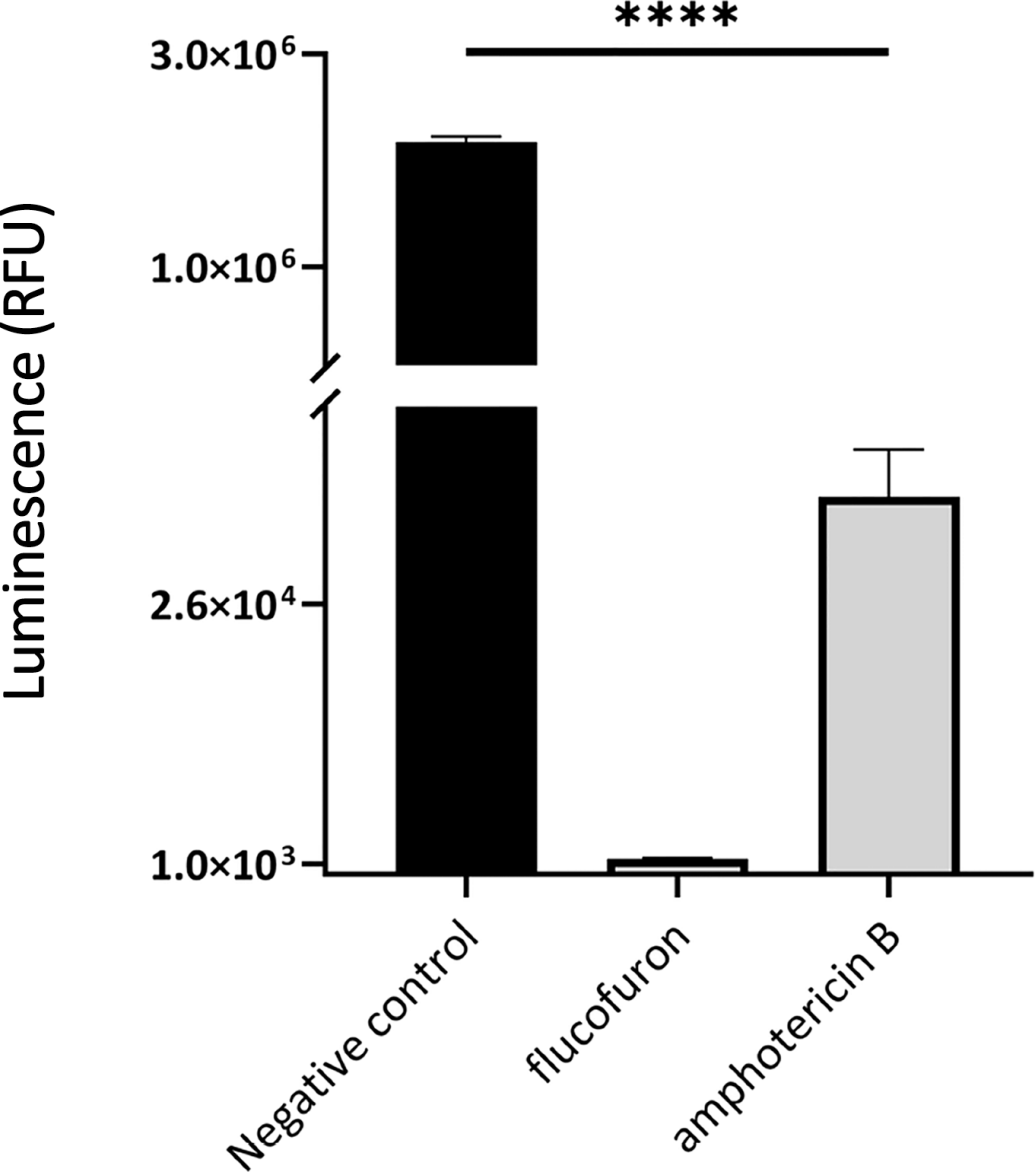
Cell Titer-Glo was employed to evaluate the ATP production. The luminescence displayed is directly related to the ATP levels produced by the amoebae. Nontreated amoebae, where the highest luminescence was observed, were used as a negative control. Treated amoebae with IC_90_ of amphotericin B (positive control) and flucofuron (evaluated compound) show inhibition of 98.33 and 99.94% relative to the negative control, respectively. Data represent the mean values of three different assays and the standard deviation (SD). An ANOVA was performed to determine the statistical differences between the treated and nontreated cells, **** *p* < 0.0001.

A mitochondrial damage event complementary to the one described earlier is the decrease in ATP levels produced in treated amoebae. For this purpose, the Cell Titer-Glo Luminescent Cell Viability Assay (Promega Biotech Ibérica, Madrid, Spain), which emits luminescence, detectable by an EnSpire Multimode Plate Reader (PerkinElmer, Madrid, Spain), proportional to the levels of ATP produced, was used. The mean values obtained in amoebae under different experimental conditions, shown in the bar graph (Figure 7), indicate a decrease in the luminescence emitted by the amoebae with the different treatments. The results revealed a reduction of 99.94% in the group treated with flucofuron and 98.33% with amphotericin B compared to the negative control. This substantial decrease was statistically validated by ANOVA (**** *p* < 0.0001), supporting the high impact of the pharmaceutical agents on mitochondrial function.

#### 1.3.5 Reactive Oxygen Species Overproduction

**Figure 8:**
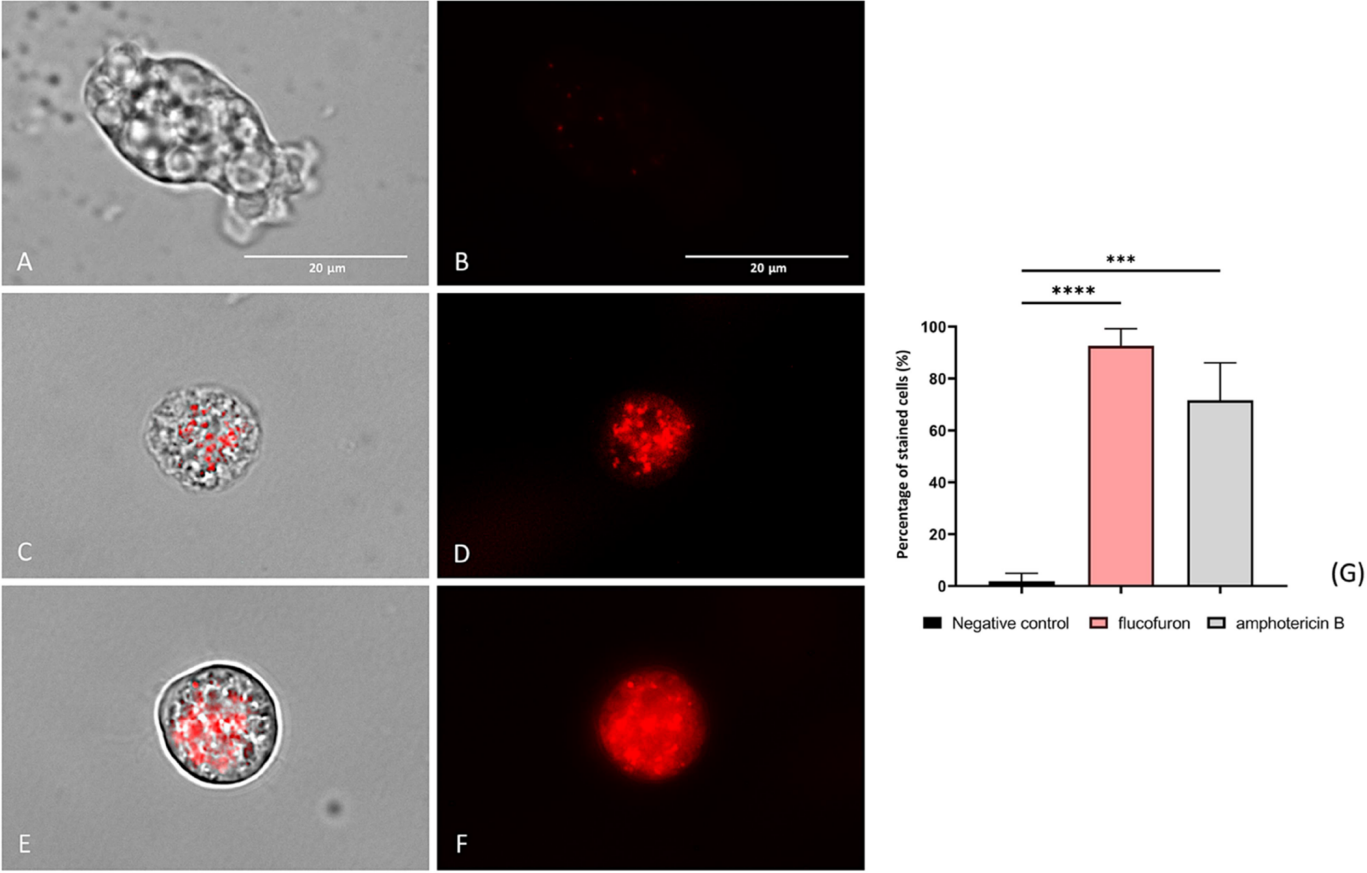
Oxidative stress was evaluated by the detection of ROS overproduction using was detected using CellROX Deep Red fluorescent kit. Nontreated amoebae were used as a negative control (A, B). Treated amoebae with flucofuron (C, D) have been tested. Treated amoebae with amphotericin B (E, F) served as a reference group to validate the results. Treated amoebae with IC_90_ of chemicals (C–F) displayed red fluorescence, due to increased ROS. This result was contrasted with an absence of fluorescence showed by negative control (A, B). Two different visible/fluorescent channels were captured simultaneously with a magnification of 100×. The EVOS M5000 Cell Imaging System, Life Technologies, Madrid, Spain, was employed to capture the images. Scale bar: 20 μm. The bar graph (G) reports the percentage of stained cells with CellROX. The data represent the average values of three separate assays, shown together with the standard deviation (SD). An ANOVA was conducted for statistical differences between treated and nontreated cells, **** *p* < 0.0001; ns, not significant. Each count involved analysis of three different images of the cell population using EVOS M5000 software tools.

Oxidative stress was measured by the elevated generation of intracellular reactive oxygen species (ROS), which are detected due to the binding of CellROX reagent (Thermo Fisher Scientific, Madrid, Spain). In the nontreated control group (Figure 8A,B), no or barely visible fluorescence was observed due to the absence of ROS accumulation. In contrast, trophozoites treated with flucofuron (Figure 8C,D) and amphotericin B (Figure 8E,F) showed red fluorescence emission, indicative of increased cellular stress due to these reactive species. Disparities between values were assessed by ANOVA, revealing significant differences (**** *p* < 0.0001; *** *p* < 0.001) when comparing treated cells with negative controls.

## 2 Discussion

In recent years, with the emergence of new diseases, the world has witnessed the rapid development of innovative tools as collaboration barriers are dismantled. As is known, several commercially available medicines have the potential to be used for more than one purpose, thus being effective in multiple ways. In addition, they would be used against neglected diseases in which there is minimal interest from pharmaceutical companies and research is limited. In the present study, the Global Health Priority Box (Medicines for Malaria Venture, Geneva, Switzerland) was provided, which contained 160 compounds suspected to be active against pathogens or vectors in order to detect anti-*Naegleria* activity. Among all of these compounds, the present study led to the identification of flucofuron as a promising molecule. Flucofuron demonstrated remarkable inhibitory effects against *N. fowleri* ATCC 30808 and 30215 with in vitro IC_50_ values of 2.58 ± 0.64 μM and 2.47 ± 0.38 on parasite viability, respectively. Moreover, the effect against the cyst stage of *N. fowleri* was also evaluated, with an IC_50_ of 0.88 ± 0.07 μM, being more effective than against the vegetative stage. The activity of the product in relation to its toxicity (83.86 ± 20.76 μM) leads to a selectivity index of 32.55 (ATCC 30808) and 33.96 (ATCC 30215), being higher compared to the effect obtained by miltefosine, giving this product a high interest.

The molecule under study is an organochlorine insecticide that has been historically used in commercial mothproofing formulations against the larvae of insects from mainly the order of Coleoptera (beetles) and Lepidoptera (butterflies and moths), which are known for their capacity to digest keratin. The pesticide activity of this molecule was based on the inhibition of the synthesis of the enzyme required to break down keratin.^22,23^ Currently, it cannot be found in any commercial products; however, its detection in sediments is still possible due to its historical use.^24^

Pesticides and insecticides have been widely used throughout history with the aim to control different pests that affect agriculture and provoke several human diseases. However, despite the bad reputation of these compounds, some studies have emerged highlighting the medicinal potential and biomedical applications of the pesticides. For instance, the dithiocarbamates, used as fungicides in agriculture, possess antiviral, antibacterial, antiparasitic, or antifungal properties that can be used in medicine.^25^ Furthermore, commercial acetohydroxyacid synthase (AHAS)-inhibiting herbicides have also been proposed to treat human fungal infections, such as the ones caused by *Cryptococcus neoformans* and *Candida albicans*, due to its high efficacy against these species and low toxicity to mammalian cells.^26^

The biological activity of the flucofuron has already been demonstrated against different bacterial strains of the *Staphylococcus aureus* and *Staphylococcus epidermidis* species.^27^ Moreover, the antiparasitic properties of this compound have also been described against juvenile and adult stages of the worms of the *Schistosoma japonicum* species.^28^

In this work, the activity of flucofuron against two type strains of *N. fowleri* was described, showing IC_50_ values around 2.50 μM in both cases. Furthermore, this compound showed low toxicity, with a CC_50_ value of 83.86 μM, meaning that the selectivity index is above 32, 10-fold times higher than the one for miltefosine, one of the reference drugs against the PAM. The evaluation of the efficacy of flucofuron was extended to the resistant stage of *N. fowleri*, showing a cysticidal IC_50_ value of 0.88 μM. In light of these results, the type of death that induces the flucofuron in the amoebae was evaluated for a better understanding of the mechanism of action of the molecule.

In broad terms, cell death processes are divided into apoptosis or programmed cell death (PCD) and necrosis. While the necrosis is referred to an accidental and passive death that involves an uncontrolled release of inflammatory cellular contents, apoptosis is described as an active and PCD process that avoids inflammation.^29^ The PCD in the amoeba of the *Naegleria* genus is characterized by DNA fragmentation, degeneration of the mitochondria, increase in ROS levels, and phosphatidylserine (PS) externalization among others.^30^ Our study concluded that some of these events can be observed after treatment of the trophozoites with the flucofuron. The PCD assays demonstrated the presence of condensed chromatin, alteration of the plasma membrane permeability, mitochondrial damage, increase in ROS levels and externalized PS levels, and DNA fragmentation detected with the TUNEL assay. These results suggest that the flucofuron induces a PCD process in the amoebae so that the appearance of inflammation and the possibility of undesired side effects are avoided.

Regarding the pharmaceutical properties, flucofuron is a small molecule with a 417.7 weight mass. Moreover, the log*P* value of this molecule is 6.2, meaning that is possess lipophilic properties. Hence, flucofuron matches some of the required properties by a molecule to cross the blood–brain barrier, the low molecular weight and a high log*P* value (lipophilic compound),^31^ which means that it can exert its action in the brain, one of the main limiting factors for the drug development against the PAM.

## 3 Conclusions

In conclusion, this work demonstrates the anti-*Naegleria* activity of the flucofuron against both trophozoites and cysts. Moreover, the PCD process induction by this molecule has also been described. These results against *N. fowleri*, coupled with its low cytotoxicity, make flucofuron a good candidate to develop new pharmaceutical agents to treat PAM. However, more studies are needed in order to understand the mechanisms of action of flucofuron and its effects on mammalians.

## 4 Materials and Methods

### 4.1 Chemicals

Molecules used were obtained from Global Health Priority Box, donated by Medicines for Malaria Venture (MMV, Geneva, Switzerland), and are a collection of 240 commercially available drugs divided into 80 that have shown activity against *Plasmodium*, the causative agent of Malaria, 80 compounds donated by Bristol Myers Squibb for the treatment of neglected diseases and drug-resistant diseases, and 80 products selected by IVCC experts against various vector species, as shown in Supporting Information S1. These compounds were initially at a concentration of 10 mM and placed in 96-well microtiter plates diluted to a concentration of 1 mM using 100% dimethyl sulfoxide (DMSO) for testing against *N. fowleri*.

Flucofuron was acquired from LGC Standards, Dr. Ehrenstorfer (LGC group, Barcelona, Spain). The molecule was chemically analyzed and certified by the producer, showing 99.7% (g/g) purity and 1% (g/g) expanded uncertainty (*U*). The product was present in the solid crystalline state and was kept at 20 °C ± 4 °C.

### 4.2 Strains and Cell Line Culture

To conduct the assays, two different strains of *N. fowleri* species were employed. These strains were obtained from the American Type Culture Collection from cerebrospinal fluid (CSF) samples, namely ATCC 30808 (Carter Kul Strain) and ATCC 30215 (Carter Nf69 Strain). This parasite was cultivated axenically in the trophozoite stage, grown in culture flasks with 2% Bactocasitone medium supplemented with 10% (v/v) fetal bovine serum (FBS), and antibiotics 0.3% penicillin G sodium salt, and 0.5 mg/mL streptomycin sulfate (Sigma-Aldrich, Madrid, Spain). Additionally, as it is a thermotolerant organism, it had to be incubated in a chamber at 37 °C. To allow its transformation into cysts, its usual medium was replaced with the MYAS medium, which, along with constant orbital agitation at room temperature for approximately 10 days, led to its encystment, enabling it to withstand unfavorable conditions that hinder its growth.^20^

For cytotoxicity assays, the J774A.1 (ATCC TIB-67) murine macrophage cell line was used. Cells were grown in culture flasks with Dulbecco’s modified Eagle’s medium (DMEM, w/v) supplemented with 10% (v/v) FBS and 10 μg/mL gentamicin (Sigma-Aldrich, Madrid, Spain) and maintained in a controlled environment at 37 °C with a 5% CO_2_ concentration to ensure optimal growth.

### 4.3 In Vitro Potential Inhibition Analysis of Global Health Priority Box against *N. fowleri*

To evaluate the activity of the 240 compounds, an initial screening was performed on our reference strain, ATCC 30808. Amoebae were incubated in a 96-well microtiter plate at a concentration of 2 × 10^5^ cells/mL for 15 min to adhere to the bottom of the wells. After this time, the compounds (comp./well) were placed on the plate diluted in bactocasitone at a final concentration of 1 μM. Untreated amoebas were used as the negative control. Finally, the alamarBlue reagent was added, indicating the level of metabolic activity and cell viability through a color change in the wells. Then, the amoebae plate was incubated at 37 °C for 48 h, and the emitted fluorescence was analyzed using an EnSpire Multimode Plate Reader (PerkinElmer, Madrid, Spain) at 570/585 nm. The obtained data were compared with those from the negative control, discarding compounds that did not meet the required values for further studies.

After the compound of interest was selected, activity assays were conducted to determine inhibitory concentrations. The protocol was similar to the one described earlier with the same amoeba concentration. The only difference was in the addition of the compounds. A 96-well microtiter plate column was used, and the drug was added at different concentrations by predilution in a deep well. Finally, the alamarBlue viability indicator was added, and the process followed the same procedure as that already described.

In addition to their activity against the infective stage, their efficacy against the resistance phase of the parasite was also demonstrated. This approach allows identification of therapeutic agents with broad-spectrum activity against *N. fowleri*, contributing to the development of more efficient treatments against this pathogen. For this purpose, the cysticidal activity of the selected compounds was evaluated. In contrast to the previous assay, compound dilutions were first prepared directly in the 96-well plate. Once the mixture was prepared, a concentration of 2 × 10^5^ cells/mL of mature cysts was added to the plate. This procedural adjustment was made due to the lack of cyst adherence to the wells. The cysts were incubated with the compound for 24 h. After this period, the plate was centrifuged, and the supernatant containing the compound was replaced with a fresh culture medium. Nontreated cysts served as the negative control. Finally, 10% of the total volume of the alamarBlue reagent was added, enabling the differentiation of nonviable cysts at higher compound concentrations and excysted trophozoites as the concentration decreased.

### 4.4 In Vitro Cytotoxic Effect of Compounds in Murine Macrophages

The evaluation of cytotoxicity for the selected products involved the use of murine macrophage cell line J774A.1 (ATCC TIB-67). Using the Countess 3 FL Automated Cell Counter (Thermo Fisher Scientific, Spain), the cells were cultured in 96-well microtiter plates at a concentration of 10^5^ cells/mL in RPMI (Roswell Park Memorial Institute, 1640) medium, supplemented with 10% (v/v) FBS under controlled conditions of 37 °C with 5% CO_2_. Once the cells were placed, the compounds previously diluted in a deep well were added to the main plate. Finally, 10% of the total volume of alamarBlue reagent was added. As previously mentioned, this reagent evaluates cell viability through colorimetric changes. Subsequently, the 96-well microtiter plate was subjected to analysis using the EnSpire Multimode Plate Reader (PerkinElmer, Madrid, Spain). The obtained data were further processed and analyzed using GraphPad Prism 9 software to determine the cytotoxic concentration (CC_50_) and subsequent calculation of the selectivity index (CC_50_/IC_50_).

### 4.5 Evaluation of PCD Events

All PCD assays were performed with a previous incubation of ATCC strain 30808 trophozoites at a concentration of 5 × 10^5^ cells/mL with the IC_90_ of flucofuron for 24 h. After incubation, different reagents for the determination of these events were added, following the manufacturer’s instructions. The results were compared with nontreated amoebae, used as a negative control, and treated with amphotericin B, considered as a positive control. In addition, the percentage of stained cells for each reagent compared to the total number of cells was analyzed from 3 images per condition and assay.

#### 4.5.1 Assessment of Apoptotic Pathways

To detect characteristic events of apoptotic cell death, various fluorescence-emitting assays were employed. For this purpose, three different assays were performed, all related to damage in the nucleic acids of the cells.

##### 4.5.1.1 Evaluation of Chromatin Condensation

One of the first processes displayed by apoptotic cells is the detection of chromatin condensation. Its determination was assessed by using a nucleus-affine dye named Hoechst 33342 (Life Technologies, Madrid, Spain). The reagent was added to the cells under each condition at a final concentration of 1 μM. Hoechst 33342 stains condensed chromatin in a blue color (350/461 nm), signaling a cellular apoptotic state.

##### 4.5.1.2 Apoptotic and Necrotic Cell Differentiation

To analyze the death stage of treated amoebae, a double staining apoptosis detection kit was employed. Annexin V Alexa Fluor 488 (Invitrogen, Thermo Fisher Scientific, Madrid, Spain) reagent was used to detect externalization of phosphatidylserine (PS), a characteristic marker in cells undergoing early stages of apoptosis, exhibiting green fluorescence. Simultaneously, propidium iodide (PI) was applied to detect late-stage apoptosis or necrosis, staining cells that emit red fluorescence. In the experimental setup, amoebae were preincubated in graduated 1.5 mL polypropylene microtubes under different conditions, some nontreated, cells treated with flucofuron, and finally, those treated with amphotericin B. After the incubation period, a single centrifugation (2500 rpm for 10 min) was performed and the supernatant was removed. The supernatant was replaced by an Annexin binding buffer, where the pellet was dissolved. Subsequently, Annexin V Alexa Fluor 488 was placed, and the mixture was incubated for 15 min in the darkness, after which PI was added. All steps and reagent concentrations were executed in accordance with the manufacturer’s guidelines. Immediately, 10 μL of the microtubes were collected, added to a Countess 3 Standard Slides, and placed in the Countess Automated Cell Counter 3 FL to differentiate live (nonfluorescence), apoptotic (green fluorescence), or death cells (red fluorescence) under different experimental conditions.

##### 4.5.1.3 DNA Fragmentation during Apoptosis

The TUNEL (Terminal deoxynucleotidyl transferase (TdT) dUTP Nick-End Labeling) assay was developed to demonstrate DNA fragmentation in treated amoebae. This assay was developed using the Click-iT TUNEL Alexa Fluor Imaging Assay (Invitrogen, Thermo Fisher Scientific, Madrid, Spain). First, *N. fowleri* trophozoites were cultured once under appropriate growth conditions. Four experimental conditions were prepared: a negative control group without treatment, nontreated cells with DNase (positive control), a flucofuron-treated group, and an amphotericin B-treated group. After the incubation period, centrifugation was performed (2500 rpm for 10 min), discarding the supernatant. The pellet was collected and added to standard gelatin-pretreated slides fixed with 4% paraformaldehyde according to the assay requirements. Once fixed, 0.3% TRITON was added to permeabilize the cells. Subsequently, the TdT enzyme, labeled with a fluorochrome-modified nucleotide, was paced to label the 3′–OH ends of the DNA fragments (green fluorescence emission, 488 nm). Samples were observed with an Echo Revolution hybrid automated microscope (Discover Echo, San Diego, CA), capturing images of the results.

#### 4.5.2 Plasma Membrane Permeability Assay

SYTOX Green (Life Technologies, Madrid, Spain) reagent was employed for the analysis of the plasma membrane permeability. Particularly, this reagent was used to identify cells with damaged or permeabilized cell membranes by emitted green fluorescence. To identify possible alterations in plasma membrane permeability, the amoebae were incubated under different conditions, and the flucofuron-treated amoebae were compared with a negative control (nontreated cells) and a positive control (amphotericin B-treated cells). For this procedure, the reagent was added to a final concentration of 1 μM. Under normal conditions, SYTOX Green has a limited ability to penetrate cells with intact cell membranes. Nevertheless, when the plasma membrane is damaged, the dye enters, resulting in specific binding to DNA and a remarkable 100-fold increase in fluorescence (504/523 nm).

#### 4.5.3 Mitochondrial Function Analysis

In this assay, the mitochondrial function of amoebae was analyzed, specifically focusing on the mitochondrial membrane potential and ATP levels. For this purpose, three distinct experimental conditions were established, including untreated amoebae (negative control), treated amoebae with flucofuron, and treated amoebae with amphotericin B. For the first aim, 10 μL of JC-1 mitochondrial membrane potential detection kit (Cayman Chemicals Vitro SA, Madrid, Spain) was added to each experimental group. This reagent would tend to form J-aggregates in the mitochondria of nontreated amoebae, producing a predominantly red fluorescence signal (∼590 nm). However, when these potentials decrease, the reagent disperses and remains in monomer form, emitting green fluorescence (∼529 nm). Hence, a decrease in the red-to-green fluorescence ratio suggests possible mitochondrial damage or dysfunction.

In addition, the Cell Titter-GLO Luminescent Cell Viability reagent (Promega Biotech Ibérica, Madrid, Spain) was used to analyze the ATP production. The mechanism is based on the detection of luminescence generated when the reagent luciferase enzyme catalyzes the reaction of luciferin with oxygen. The luminescence obtained was measured using an EnSpire Multimode Plate Reader (PerkinElmer, Madrid, Spain). Luminescence emitted by treated amoebae was compared with the negative control group.

#### 4.5.4 Evaluation of Oxidative Stress

The presence of ROS overproduction in amoebae was conducted using the CellROX Deep Red (Invitrogen, Thermo Fisher Scientific, Madrid, Spain). The experiment included nontreated amoebae (negative control) and treated amoebae with the compounds flucofuron and amphotericin B (positive control). The reagent was added at a final concentration of 5 μM. In addition, the potential presence of intracellular ROS was reflected by the emission of red fluorescence at 644/665 nm.

### 4.6 Data Analysis

All experiments conducted in this article were performed in triplicate, and the mean and standard deviation were obtained. GraphPad Prism 9.0 (GraphPad Software, CA, USA) was utilized for data analysis. Inhibitory concentrations were determined through a nonlinear regression, and distinctions between values were examined employing a one-way analysis of variance (ANOVA). Results are expressed as the mean ± standard deviation (SD) derived from the triplicate experiments. Statistically significant differences were considered when the mean difference was *p* < 0.05.

## Supporting Information Available

Global Health Priority Box details; GHPB plate map - ZND plate, VEC plate, MB2 plate (ZIP)

## Abbreviations

AHAS: acetohydroxyacid synthase
ANOVA: one-way analysis of variance
ATCC: American Type Culture Collection
BBB: blood–brain barrier
BSL-3: biosafety level 3
CC_50_: cytotoxic concentration 50
CNS: central nervous system
DMEM: Dulbecco’s modified Eagle’s medium
DNA: deoxyribonucleic acid
FBS: fetal bovine serum
IC_50_: inhibition concentration 50
IC_90_: inhibition concentration 90
JC-1: 5,5′,6,6′-tetrachloro-1,1′,3,3′-tetraethylbenzimidazolocarbocyanine iodide
MYAS: malt extract, yeast extract, and amoebae saline
MMV: Medicines for Malaria Venture
PAM: primary amoebic meningoencephalitis
PCD: programmed cell death
PCR: polymerase chain reaction
PI: propidium iodide
ROS: reactive oxygen species
SD: standard deviation
SI: selectivity index
TUNEL: terminal deoxynucleotidyl transferase (TdT) dUTP nick-end labeling
